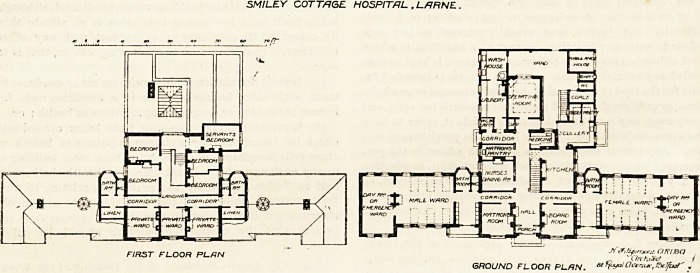# The Cottage Hospital at Larne, Antrim

**Published:** 1903-07-18

**Authors:** 


### THE COTTAGE HOSPITAL AT LARNE, ANTRIM.

THIS building consists of a central block and two wings, and in its general design shows considerable knowledge of the planning of small hospitals, being a distinct advance on the average of such institutions. The entrance is in the centre of the main elevation. On the right is the board-room and on the left is the matron's room. Behind these is the connecting corridor which we fear may be rather dark unless the room opposite the bath-room, which looks like a store-room, have no inner wall, which indeed seems to be the case. Opposite this room are the bath-room and closet. The latter is incorporated with the block, and it must be deficient in light and air. It would have been infinitely better to have projected the bath-room and closet from the north corner of the day-room, and thus have easily obtained a cross-ventilated passage between the sanitary block and the main. This alteration should be made without delay.

The dormitories are for six beds each. There are three windows on one side and only two on the other. It is not

SMILEY COTTAGE HOSPITAL, LARNE.

FIRST FLOOR PLAN

GROUND FLOOR PLAN.

N. Fitzsimons ARIBA Architect 86 Royal Avenue, Belfast easy to see why there were not three on both sides. With the removal of the bath-room and closet to the position we have indicated, the spaces now occupied by them could be used as nurses' duty-rooms.

The kitchen is on the north side of the corridor and is balanced by the nurses' dining-room, between them being the staircase and passage. Further north are the dispensary, the pantry, scullery, laundry, and other offices. The operating-room is also here and is contiguous to the laundry. It has a good north window and a large skylight.

The first floor contains three private wards for one bed each, five bedrooms for the staff, linen-rooms and bath-rooms.

The wards are warmed on the correct system of open fire-places and hot water radiators as auxiliaries.

The outside walls are hollow, and are built of red brick and have stone dressings. The upper story is pebble dashed, and the gables are constructed of half timber work. The roofs are covered with Brosley tiles. The south elevation is decidedly pleasing.

The total cost of the hospital will be about £5,000; and the district owes it solely to the munificence of Mr. Smiley who has also given a free site.

The total accommodation is for 20 beds.

The architect is Mr. Fitzsimons of Belfast, and the contractor is Mr. James Ferris.

## Figures and Tables

**Figure f1:**